# Hyper-Immunoglobulin E (IgE) Syndrome: A Diagnostic Dilemma

**DOI:** 10.7759/cureus.42729

**Published:** 2023-07-31

**Authors:** Omaira Tejada Amaro, Victor N Oboli, Smita Kumar

**Affiliations:** 1 Pediatrics, New York City Health and Hospitals/Lincoln, Bronx, USA; 2 Allergy and Immunology, New York City Health and Hospitals/Lincoln, Bronx, USA

**Keywords:** asthma, osteomyelitis, sinopulmonary infections, elevated serum ige, eosinophilia, recurrent infections, atopic dermatitis, hyper immunoglobulin e syndrome

## Abstract

Hyper-immunoglobulin E (IgE) syndrome (HIES) is an immunodeficiency syndrome characterized by atopic dermatitis, recurrent skin abscesses, and sinopulmonary infections with elevated serum IgE. In addition, patients also present with other skeletal and non-immune symptoms.

We present a six-year-old boy with severe atopic dermatitis, multiple food allergies, mild asthma, and recurrent sinopulmonary infections, who presented to the ER with left ankle pain, fever, and inability to bear weight. Physical examination showed generalized eczematous lesions, significant left ankle ecchymosis, swelling, and tenderness. Investigations were pertinent for leukocytosis with neutrophilia and markedly elevated IgE levels with normal IgM, IgG, and IgA levels. HIES genetic panel was negative. MRI with contrast of the affected limb was consistent with osteomyelitis that responded to antimicrobial therapy. This case highlights a diagnostic challenge for allergists and clinicians when evaluating patients with severe atopic dermatitis, recurrent infections, and markedly elevated serum IgE without positive genetic results.

## Introduction

HIES is a clinical condition comprising atopic dermatitis, recurrent skin abscesses and sinopulmonary infections, and high IgE levels. Patients also present with other skeletal and non-immune symptoms. Individuals with atopic dermatitis and markedly elevated IgE with atypical clinical features should be evaluated for possible HIES [1].

## Case presentation

**Figure 1:**
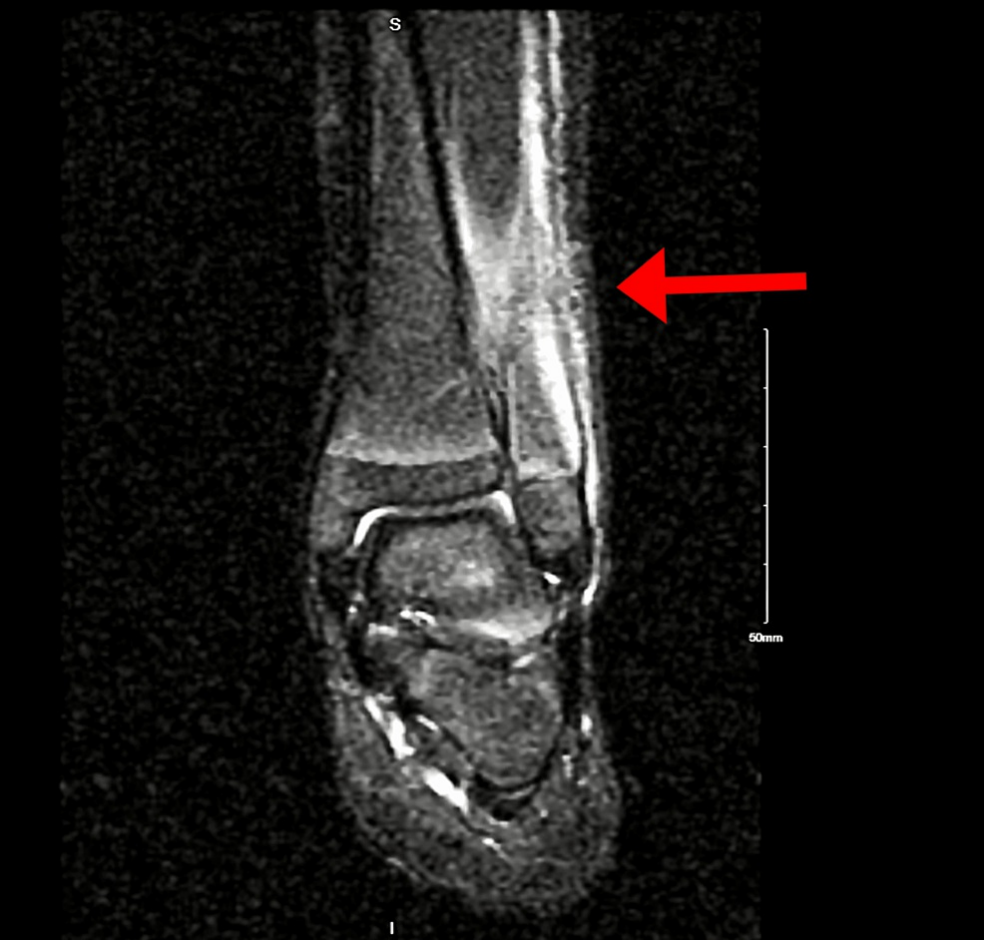
MRI of the left lower extremity showing subperiosteal collection (red arrow) and changes consistent with osteomyelitis

A six-year-old boy with a history of severe atopic dermatitis, multiple food and drug allergies, mild asthma, and recurrent sinopulmonary and cutaneous infections, including methicillin-resistant *Staphylococcus aureus* (MRSA) infection (on dupilumab treatment for the last two years), presented to the ED with a five-day history of left ankle pain, high-grade fever, and inability to bear weight. There was no history of fever, recent trauma, puncture wounds, or recent skin infection prior to presenting to the ED. Physical examination showed hyperextensible joints, generalized eczematous lesions, significant left ankle ecchymosis, swelling, and tenderness with no dysmorphic facial features. Laboratory studies were pertinent for leukocytosis with neutrophilia, elevated C-reactive protein and erythrocyte sedimentation rate, and markedly elevated IgE levels (31960 KU/L; reference range: <100 KU/L). IgM, IgG, and IgA levels were normal, and the blood culture was negative. HIES genetic panel (DOCK8, SPINK5, STAT3, TYK2) was negative. Left ankle X-ray showed periarticular soft-tissue swelling. MRI with and without contrast of the left ankle showed findings consistent with osteomyelitis (Figure 1). He was admitted and treated with intravenous antibiotics (vancomycin and cefepime and later changed to ceftaroline, clindamycin, and daptomycin) and supportive care. Surgery was consulted, and he had an incision and drainage of an abscess that cultured “MRSA.” On clinical improvement, he was discharged home on oral linezolid to complete four weeks of therapy, continued MRSA decolonization regimen, and advised follow-up in allergy and genetic clinics.

## Discussion

HIES is a primary immune deficiency syndrome composed of immune and non-immune features inherited via autosomal dominant and autosomal recessive patterns. The most commonly affected genes are STAT3, DOCK8, SPINK5, and TYK2, but several other genes have been associated with the clinical syndrome [1-3]. Autosomal dominant STAT3 mutations have been commonly reported [4]. STAT3 is the gene responsible for Th17 cell development and T cell-mediated responses [1]. The initial presentation is usually a newborn pustular and eczematoid rash in the first month of life [4]. Additional manifestations are coarse facial features, musculoskeletal anomalies (bone fractures, hyperextensible joints, osteoporosis, scoliosis), gastrointestinal symptoms (eosinophilic esophagitis, gastroesophageal reflux), high-arched palate, and retained primary teeth [1,3]. Patients are free of atopy, such as asthma, allergic rhinitis, food allergy, and anaphylaxis [2]. Despite having the clinical manifestations of atopy and certain features of HIES, our patient tested negative for HIES on the genetic panel. This makes diagnosing HIES in patients with features of atopy and elevated IgE levels challenging. The two most common laboratory findings of HIES are elevated serum IgE above 2000 IU/ml and eosinophilia, which can be present since birth [4]. The total white blood cells and other immunoglobulins are usually within normal limits. Genetic testing can aid in diagnosing HIES, especially STAT3 HIES. The differential diagnoses such as immunodeficiencies, Wiskott-Aldrich and Omenn syndromes, must be ruled out [1].

Treatment focuses on symptom control and early antimicrobial therapy. In addition, treatment targets the most common bacteria implicated: *Staphylococcus aureus*, *Streptococcus pneumoniae*, and *Haemophilus influenzae* [4]. Mortality increases with recurrent pyogenic pneumonia. Physiological changes to the lungs lead to the formation of pneumatoceles and bronchiectasis. Further studies are underway to determine the benefit of using hematopoietic stem cell transplant therapy in these patients [2].

## Conclusions

Our patient had features of HIES but without dysmorphic features or positive genetic results, which poses a challenge in diagnosing HIES. Therefore, in patients with a history of severe atopic dermatitis and recurrent infections in early childhood with elevated serum IgE, prompt and accurate diagnosis requires a high index of suspicion for HIES, especially if dysmorphic features are present. When in doubt, affected children should be referred to the allergist for further evaluation.
